# Transcriptional Activation of *TINF2*, a Gene Encoding the Telomere-Associated Protein TIN2, by Sp1 and NF-κB Factors

**DOI:** 10.1371/journal.pone.0021333

**Published:** 2011-06-23

**Authors:** Zhong-Tao Xin, Kathryn A. Carroll, Naveen Kumar, Kui Song, Hinh Ly

**Affiliations:** Department of Pathology and Laboratory Medicine, Emory University School of Medicine, Atlanta, Georgia, United States of America; University of South Florida , United States of America

## Abstract

The expression of the telomere-associated protein TIN2 has been shown to be essential for early embryonic development in mice and for development of a variety of human malignancies. Recently, germ-line mutations in *TINF2*, which encodes for the TIN2 protein, have been identified in a number of patients with bone-marrow failure syndromes. Yet, the molecular mechanisms that regulate *TINF2* expression are largely unknown. To elucidate the mechanisms involved in human *TINF2* regulation, we cloned a 2.7 kb genomic DNA fragment containing the putative promoter region and, through deletion analysis, identified a 406 bp region that functions as a minimal promoter. This promoter proximal region is predicted to contain several putative Sp1 and NF-κB binding sites based on bioinformatic analysis. Direct binding of the Sp1 and NF-κB transcription factors to the TIN2 promoter sequence was demonstrated by electrophoretic mobility shift assay (EMSA) and/or chromatin immunoprecipitation (ChIP) assays. Transfection of a plasmid carrying the Sp1 transcription factor into Sp-deficient SL2 cells strongly activated TIN2 promoter-driven luciferase reporter expression. Similarly, the NF-κB molecules p50 and p65 were found to strongly activate luciferase expression in NF-κB knockout MEFs. Mutating the predicted transcription factor binding sites effectively reduced TIN2 promoter activity. Various known chemical inhibitors of Sp1 and NF-κB could also strongly inhibit TIN2 transcriptional activity. Collectively, our results demonstrate the important roles that Sp1 and NF-κB play in regulating the expression of the human telomere-binding protein TIN2, which can shed important light on its possible role in causing various forms of human diseases and cancers.

## Introduction

Telomeres are complex nucleoprotein structures at chromosome ends that function to prevent chromosome fusions and genomic instability (reviewed in [1]). Mammalian telomeres consist of repetitive (T_2_AG_3_)n DNA sequence and associated proteins that are collectively known as the shelterin complex. The shelterin complex consists of at least six proteins TRF1, TRF2, Rap1, TIN2, POT1, and TPP1 that are required for telomere protection and length control (reviewed in [2]). The first of these proteins, the telomere-repeat binding factor 1 (TRF1), was isolated based on its ability to bind double-stranded TTAGGG repeats [3], [4], followed soon after by the identification of its paralog TRF2 [5], [6]. TRF1-interacting nuclear protein 2 (TIN2) and Rap1 were found through yeast two-hybrid screens for proteins that could interact with TRF1 and TRF2, respectively [7], [8]. Finally, a search for TIN2-interacting proteins yielded TPP1 [9], and POT1 was pulled out based on sequence homology to similar telomere-protecting proteins in unicellular eukaryotes [10].

TIN2 is an important component of the shelterin complex as it binds directly to the double-stranded telomeric DNA binding proteins TRF1 and TRF2 and indirectly interacts with the single-strand telomeric DNA binding protein POT1 via the intermediary protein TPP1 [7], [9]. Overexpression of TIN2 can shorten telomere length in telomerase-positive human cells, similar to the effect of overexpressing the TRF1 protein, implicating both proteins as a negative regulators of telomere length [7]. In contrast, TIN2 depletion via shRNA disrupts TRF1 and TRF2 binding and causes cell death, even in the absence of p53 function [11], [12]. While TIN2 remains at telomeres in growth-arrested cells, it appears to form large complexes outside the telomeres, implying that TIN2 may play other important roles in mammary epithelial differentiation [13], a hypothesis supported by the identification of a novel isoform of TIN2 which can localize to the nuclear matrix [14]. Furthermore, knock-out of TIN2 in a mouse model results in early embryonic lethality prior to embryonic day 7.5 in a telomerase-independent manner [15]. Such important roles of TIN2 have prompted several laboratories to screen patients with degenerative bone-marrow failure syndromes that are known to be associated with telomere dysfunction for natural mutations in this gene. These efforts have led to the identification of several natural sequence variations in the *TINF2* gene [16], [17], [18], [19]. However, the specific mechanisms through which these mutations may act to affect disease pathology remain unknown.

In addition to the experimental alterations in TIN2 protein levels, which clearly demonstrate that TIN2 level changes can disrupt telomere end structure and result in cell distress and/or death, several studies have shown that the changes in the endogenous expression level of several telomere-binding proteins (including TIN2) may be associated with various forms of human cancer [20], [21], [22], [23], [24]. As over 90% of cancers have also been shown to upregulate the catalytic component of the telomere-elongating enzyme telomerase, more careful studies of the transcriptional regulation of the telomere-binding proteins that have been directly implicated in telomere maintenance are warranted. Furthermore, it has recently been shown that the telomere-binding protein hRap1 and the transcription factor NF-κB positively regulate each other through a feed-forward loop [25]. To this end, we have for the first time characterized both the *cis*-elements and *trans*-acting factors that regulate the transcription of the human *TINF2* gene, which encodes for the TIN2 protein. This comprehensive examination of the transcriptional regulation of the *TINF2* gene will shed important light on the role(s) this gene plays in the pathogenesis of human hematological diseases and cancer.

## Results

### Cloning and mapping the *TINF2* promoter

**Figure 1:**
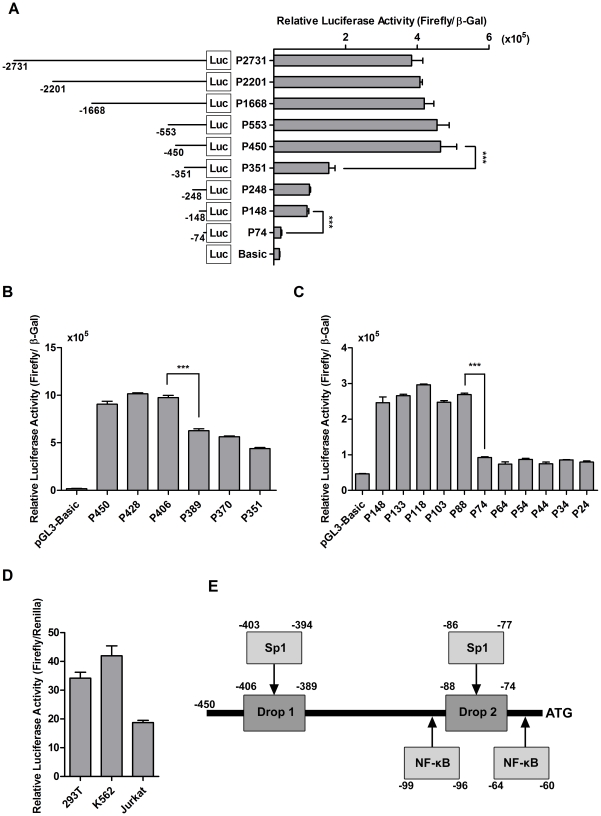
Luciferase assay analysis of *TINF2* promoter truncations defines two significant drops in promoter activity. **A**: The name of each TIN2 reporter construct was assigned according to the 5′-end nucleotide numbers of the promoter sequences inserted upstream of the ATG initiation codon. Basic refers to the pGL3-Basic vector. For each transfection, the firefly luciferase activity was normalized to the β-galactosidase activity expressed from a co-transfected β-galactosidase expression vector. The means from three independent experiments are shown for each construct; *bars*, SD. (***p<0.001). **B**: Finer promoter mapping of the region between −450 and −351, expressed as in panel A. **C**: Finer promoter mapping of the region between −148 and −24, expressed as in panel A. **D**: Verification of the functionality of the *TINF2* promoter construct in various cell lines. For each transfection, the firefly luciferase activity was normalized to the *Renilla* activity expressed from the co-transfected pRL-CMV expression vector and expressed as in panel A. **E**: Schematic of the core *TIN2* promoter construct showing putative Sp1 and NF-κB binding sites predicted using bioinformatic tools. “Drop 1” and “Drop 2” refer to the drops in luciferase reporter activity shown in panel A.

To study promoter activation, the region located immediately upstream of the initiation codon of the human *TINF2* gene was cloned from genomic DNA of HeLa cells by polymerase chain reaction into the pGL3-Basic luciferase reporter vector. The sequence was confirmed by DNA sequencing, which was found to completely match the corresponding TIN2 promoter proximal sequence on chromosome 14 genomic contig (NT 026437) reported by The Human Genome Project. A comparison of luciferase activity in extracts prepared from 293T cells transfected with the P2731 plasmid that contains the luciferase reporter gene under the control of the longest sequence (∼2.7 kb) of the *TINF2* promoter proximal region with those transfected with the pGL3-Control vector that contains the viral SV40 promoter-driven luciferase gene showed that the *TINF2* promoter functioned as a reasonably strong promoter (Fig. 1A).

To better define the *cis*-acting sequences responsible for the transcriptional activation of *TINF2*, a series of successive 5′ deletions of *TINF2* promoter sequence were generated based on the available unique restriction enzyme sites on the P2731 vector (Fig. 1A). These were again assayed for their ability to produce luciferase reporter activity in a series of transient transfection reactions of the individual plasmids into 293T cells. All constructs that contain large regions of the *TINF2* promoter sequence (i.e. P2201, P1668, P553, and P450) produced levels of luciferase activity that are comparable to those generated by the P2731 construct (Fig. 1A). In contrast, a significant drop in luciferase activity (∼3 fold, p<0.001) was observed with constructs containing *TINF2* promoter sequence of less than 351 base pairs (i.e. P351, P248, and P148). Further deletion of the *TINF2* promoter region (P74) resulted in a second significant drop in reporter activity to the basal levels of luciferase activity generated by the promoter-less pGL3-Basic construct (Fig. 1A). Collectively, these results indicate that sequences responsible for *TINF2* promoter regulation are contained within a core region encompassing 450 bps upstream of the translational initiation site of the *TINF2* gene.

Further successive deletion analysis of the P450 proximal promoter construct generated two additional constructs (P389 and P370) that produced similar levels of luciferase activity to P351 (Fig. 1B), strongly suggesting that elements responsible for activating the *TINF2* gene are primarily situated between positions −406 and −389, thus designating a minimal promoter of 406 base pairs. We also created a series of 10 deletion constructs spanning the sequence from position −148 to −74 (Fig. 1C). When these deletion constructs were transfected into 293T cells, robust luciferase expression was observed with P148, as well as with all constructs that contain longer than 88 bps of the *TINF2* promoter sequence (Fig. 1C, P148, P133, P118, P103, and P88). In contrast, all constructs with less than 74 bps of promoter proximal sequence (P74, P64, P54, P44, P34 and p24) showed levels of the luciferase activity that were comparable to that of the negative control vector pGL3-Basic (Fig. 1C, Vector). These observations suggest that there is another important *cis*-acting element located between positions −88 and −74. Taken together, these promoter mapping experiments revealed two major regions within the *TINF2* promoter proximal region located between positions −(406−389) and positions −(88−74) that contain essential transcriptional activating elements of the *TINF2* gene in 293T cells.

### Prediction of putative regulatory elements within the *TINF2* promoter and validation of the cloned *TINF2* promoter sequences in different cell lines

Sequence analysis revealed that the *TINF2* promoter lacks the conventional TATA and CAAT boxes as predicted for many of the GC-rich promoters, such as that of the *TINF2* gene. When various bioinformatics methods (TESS, Genomatix, and Gene Regulation search programs) were used, they all predicted a number of potential transcription factor binding sites on the core P450 *TINF2* promoter sequence, including potential binding sites for Sp1, AP-2, and NF-κB (data not shown). The abundance of these sites suggests the possibility that *TINF2* expression may be subject to multiple levels of control and be regulated by different factors in different cellular contexts. As such, we validated the functionality of the core *TINF2* promoter by transfecting the P450 construct into various cancer cell lines, including human leukemia K562 cells and Jurkat cells (Fig. 1D). As shown in Fig. 1E, a few of the putative transcription factor binding sites fall near the two regions where we have observed significant drops in luciferase reporter activity (Fig. 1B, 1C), allowing us to focus on these specific sequence elements.

### Binding of Sp1 to TINF2 promoter *in vitro*

**Figure 2:**
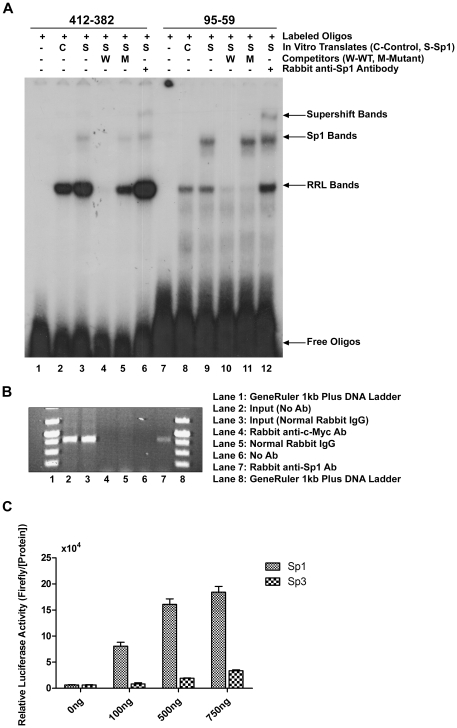
Sp1 binds to putative binding sequences in the *TIN2* promoter *in vitro* and *in vivo.* **A**: EMSA showing the ability of Sp1 to bind to its two predicted sites *in vitro.* Sp1 protein was *in vitro* translated using the rabbit reticulocyte lysate (RRL) system and incubated with end-labeled DNA oligos encompassing the putative binding sites. These complexes (lanes 3 and 9) could be competed away with unlabeled wild-type oligos (lanes 4 and 10), but not with a mutant form (lanes 5 and 11). Furthermore, the complexes could be super-shifted using an anti-Sp1 antibody (lanes 6 and 12). **B**: The ability of Sp1 to bind to the endogenous *TINF2* promoter *in vivo* was shown using ChIP. The 407 base pair fragment could be amplified from a reaction including the anti-Sp1 antibody (lane 7), but not from reactions containing anti-c-Myc, normal IgG, or no antibody (lanes 4–6). **C**: Sp1 is the major Sp transcription factor that can activate the *TINF2* promoter. *Drosophila melanogaster* SL2 cells were co-transfected with the minimal promoter reporter construct pGL3-P406 and various amounts of expression vectors encoding either Sp1 or Sp3. For each transfection, the firefly luciferase activity was normalized to the total protein concentration. The means from three independent experiments are shown; *bars*, SD.

To determine whether Sp1 can bind to its putative binding sites in the *TINF2* promoter, an electromobility shift assay (EMSA) was performed using each putative site as a DNA probe and the recombinant Sp1 protein that could be abundantly and correctly translated in the rabbit reticulocyte lysate (data not shown). A specifically strong Sp1-shifted DNA band was observed with a DNA oligo containing the putative Sp1-binding site spanning the GC-rich motif at positions −(88−74) (Fig. 2A, lane 9). This Sp1-shifted band could be super-shifted by the addition of an antibody against the Sp1 protein (Fig. 2A, lane 12). Using a second DNA oligo representing another potential Sp1-binding site at positions −(406−389) and a similar amount of the *in vitro* translated Sp1 protein, a weak but consistent Sp1-shifted band was observed (Fig. 2A, lane 3) that could also be super-shifted by the addition of the anti-Sp1 antibody (Fig. 2A, lane 6), demonstrating that Sp1 can bind with relatively high affinity to both of these sequences *in vitro*.

The specificity of the interactions was demonstrated by the disappearance of the shifted bands when 100-fold molar excess of unlabeled wild-type probe was added to the reactions as competitors (Fig. 2A, lanes 4 and 10). In contrast, no competition was observed when using unlabeled probes that contain 2-bp substitutions of conserved core nucleotides mutations in these putative Sp1-binding sequences (Fig. 2A, lanes 5 and 11). Further Sp1-binding specificity was demonstrated by the failure to generate a shifted complex with any of these DNA oligos with the *in vitro* translated AP-2 protein or with the radiolabeled DNA probes containing 2-bp substitution mutations in these two motifs using similar gel shift conditions (data not shown). A strong band located between the unbound oligos and the Sp1-shifted oligos (i.e. the RRL binding band) was observed in most of the reactions, even in a reaction when the pcDNA3.1 control vector (Fig. 2A, C-Control) was used in the rabbit reticulocyte lysates (RRL), suggesting that some endogenous transcription factors in the RRL can also bind to the radiolabeled DNA probes. Collectively, these data show that Sp1 can indeed specifically bind *in vitro* to two DNA elements located within positions −(406−389) and −(88−74) of the *TINF2* promoter proximal region.

### Binding of endogenous Sp1 proteins to the native *TINF2* promoter in cells

Chromatin immunoprecipitation (ChIP) was next used to determine whether endogenous Sp1 protein localizes to the native *TINF2* promoter. Sheared DNA from 293T cells was immunoprecipitated using antibodies specific to the large subunit of Sp1, c-Myc, or control IgG. *TINF2* proximal promoter DNA was detected by PCR using primers that amplify a 407 bp product. Figure 2B shows that anti-Sp1 antibody could effectively and specifically precipitate proteins bound to the *TINF2* promoter proximal region encompassing the minimal 406 base pair promoter, which contains the two putative Sp1-binding sites (Fig. 2B, lane 7), whereas anti-c-Myc antibody and non-specific IgG antibody failed to precipitate any protein-DNA complexes (Fig. 2B, lanes 4 and 5). These findings clearly demonstrate that Sp1 indeed can bind to the *TINF2* promoter proximal region in 293T cells.

### Sp1 is a transcriptional activator of the *TINF2* promoter

Sp1 and the related Sp3 are major factors of the Sp family of transcription factors which can serve redundant roles in cells. They are expressed in most mammalian cells except in the *Drosophila melanogaster* SL2 cells, which allows for a convenient means to determine whether Sp1 or Sp3 specifically activates the *TINF2* promoter in cell culture. To do this, we co-transfected SL2 cells with the minimal *TINF2* promoter-driven luciferase reporter construct P406 and either the *Drosophila* expression vector pPac-Sp1 or pPac-Sp3. As shown in Figure 2C, upon addition of the Sp1 transcription factor, we observed a very strong and dose-dependent induction of luciferase reporter expression. In contrast, while Sp3 appeared to also able to active *TINF2* promoter-driven luciferase activity, the effect was much weaker as compared to that obtained with the Sp1 transcription factor. These data strongly argue that Sp1, rather than Sp3, is the major transcriptional activator of *TINF2*.

### NF-κB binds to and transactivates the *TINF2* promoter

**Figure 3:**
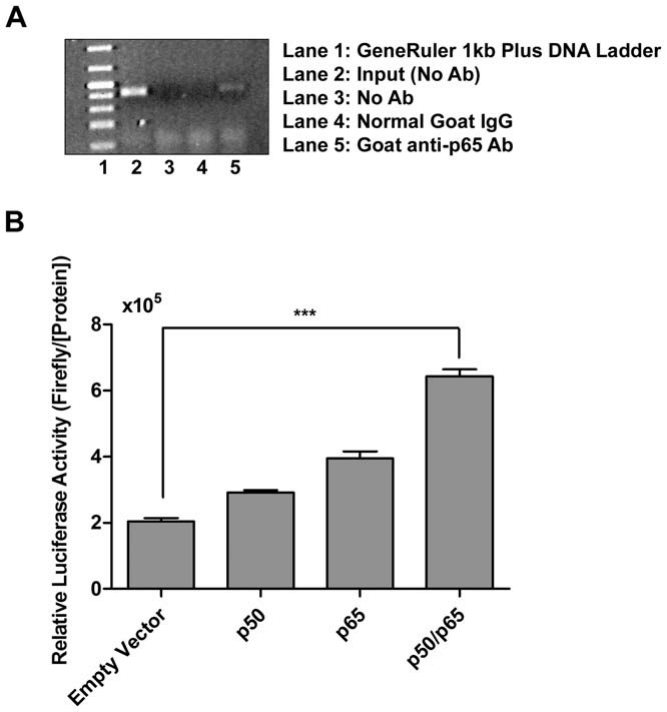
NF-κB can bind to and activate the *TINF2* promoter. **A**: The ability of NF-κB to bind to the endogenous *TINF2* promoter *in vivo* was shown using ChIP. The 407 base pair fragment could be amplified from a reaction including the anti-p65 antibody (lane 5), but not from reactions containing no antibody or normal IgG (lanes 3 and 4). **B**: The ability of NF-κB to activate the minimal *TINF2* promoter was verified by co-transfection of vectors encoding p50 and/or p65 protein and pGL3-P406 into NIH 3T3 (p50^−^/p65^−^) cells. For each transfection, the firefly luciferase activity was normalized to the total protein concentration. The means from three independent experiments are shown; *bars*, SD. (***p<0.001).

Various bioinformatic methods predicted two potential NF-κB binding sites residing near position −(82−78) (Fig. 1E). A chromatin immunoprecipretation (ChIP) assay was carried out using an antibody to the major p65 subunit of the NF-κB complex and lysate prepared from the 293T cells as described in the Materials and Methods section. We observed binding of endogenous NF-κB to the native *TINF2* promoter proximal region *in vivo* (Fig. 3A, lane 5). In order to validate these findings, we co-transfected the minimal *TINF2* promoter-driven luciferase reporter plasmid P406 with plasmids encoding the two major NF-κB components p50 and p65, either individually or simultaneously, into NF- κB knockout NIH 3T3 (p50^−^/p65^−^) cells. Relative to a control lysate of cells transfected with an empty expression vector, luciferase activities were found to be minimally increased when cells were transfected with either the p50 or p65 components but were significantly increased when plasmids containing both factors were co-transfected into cells (Fig. 3B). These data suggest that, in addition to Sp1, NF-κB also serves an important role in regulating *TIN2* promoter activity.

### Mutating the putative binding sites abolishes Sp1 and NF-κB transcriptional activation of the *TINF2* promoter

**Figure 4:**
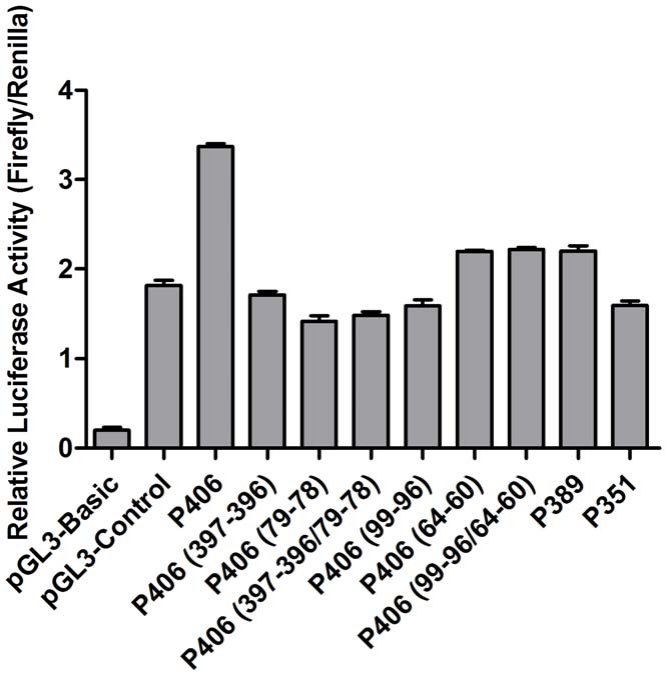
Mutating the putative binding sites abolishes Sp1 and NF-κB transcriptional activation of the *TINF2* promoter. **A**: Mutating core consensus nucleotides in predicted Sp1 or NF-κB binding sites (see Materials and Methods) results in reduced *TINF2* promoter-driven luciferase activity in a minimal promoter context. For each transfection, the firefly luciferase activity was normalized to the *Renilla* activity expressed from the co-transfected pRL-CMV expression vector. The means from three independent experiments are shown; *bars*, SD. (p<0.001). **B**: Mutating core consensus nucleotides in the predicted Sp1 binding site at position −(88−74) results in reduced *TINF2* promoter-driven luciferase activity as compared to wild-type control. Results are shown as in panel A. (***p<0.001).

In order to determine whether the predicted Sp1 and NF-κB binding sites are important for transcriptional activation of the *TINF2* promoter, core consensus nucleotides (see Materials and Methods) were mutated in the P406 plasmid which contains the minimal 406 bp promoter sequence. When transfected into HEK293 cells, plasmids carrying single mutations in either Sp1 or NF-κB binding sites showed significantly decreased levels of *TINF2* promoter-driven luciferase activity relative to the P406 wild-type construct (Fig. 4). This reduction in promoter activation is consistent with both our ChIP and EMSA data (Fig. 2A, 2B, and 3A) and supports the hypothesis that not only can Sp1 and NF-κB bind to and transactivate the *TINF2* promoter, but they do so by binding to the specific sites predicted using bioinformatics programs. However, as plasmids carrying double mutations in both Sp1 or NF-κB binding sites do not show synergistic reductions in luciferase activity (Fig. 4), the different putative transcription factor binding sites may serve redundant roles or be utilized under different cellular contexts.

### Pharmacological inhibitors can interfere with Sp1- and NF-κB-activated TINF2 transcription

**Figure 5:**
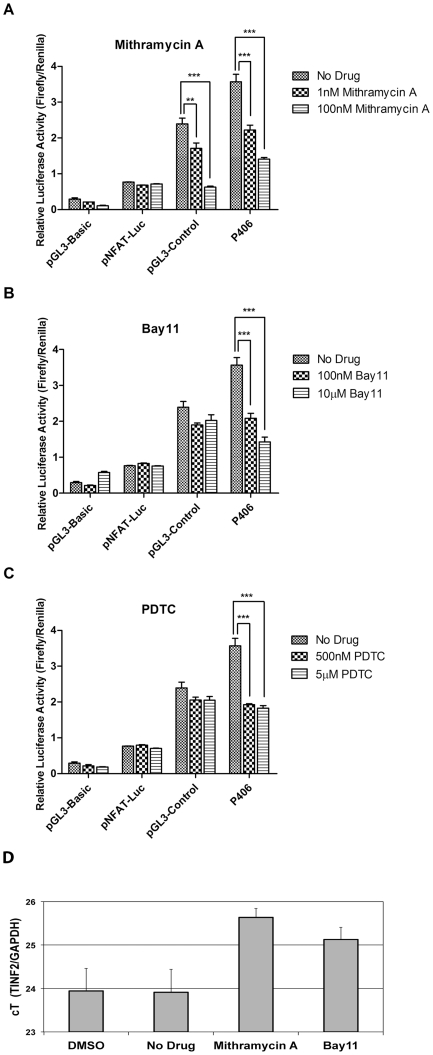
Pharmacological inhibitors can interfere with Sp1- and NF-κB-mediated *TINF2* promoter activation. **A**: Mithramycin A, an Sp1 inhibitor, reduces *TINF2* promoter-driven luciferase activity. 293T cells are transfected with either the promoter-less pGL3-Basic plasmid, the NFAT-responsive pNFAT-Luc plasmid, the SV40 promoter-containing pGL3-Control plasmid, or the minimal TIN2 promoter P406 plasmid and incubated with the indicated concentrations of drug for 24 hours. For each transfection, the firefly luciferase activity was normalized to *Renilla* luciferase activity expressed from a co-transfected *Renilla* luciferase expression vector. The means from three independent experiments are shown; *bars*, SD. (**p<0.01, ***p<0.001). **B**: Bay11-7082, an NF-κB inhibitor, reduces *TINF2* promoter-driven luciferase reporter activity. Cells are processed and values expressed as in panel A. (***p<0.001). **C**: PDTC, an NF-κB inhibitor, reduces *TINF2* promoter-driven luciferase reporter activity. Cells are processed and values expressed as in panel A. (***p<0.001). **D**. Mithramycin A and Bay11-7082 (Bay11) reduce endogenous *TINF2* gene expression. The levels of *TINF2* mRNA were normalized to those of the housekeeping *GAPDH* gene. The means from three independent experiments are shown.

Various pharmacological inhibitors exist which have been shown to interfere with transcription factor binding and/or activation of their target promoters. Mithramycin A, a GC-specific DNA-binding drug [26] prevents binding of Sp1 to its consensus GC-rich binding sites [27]. When added to 293T or HEK293 cells transfected with the minimal promoter reporter construct P406 or control plasmid constructs (pGL3-Basic, pNFAT-Luc, or pGL3-Control), Mithramycin A was able to specifically and significantly reduce the reporter activity of both the *TINF2* promoter and the SV40 promoter-containing pGL3-Control plasmid, both of which contain Sp1 binding sites. No effect was observed in chemically treated cells transfected with the negative control vectors pGL3-Basic or pNFAT-Luc (Fig. 5A). Similar results were obtained in Mithramycin A-treated HEK293 cells that were transfected with a similar set of plasmids (data not shown). Bay11-7082 (Bay 11), which has been shown to irreversibly inhibit NF-κB activation by blocking TNF-α-induced phosphorylation of IκB [28], also specifically and significantly reduced NF-κB-mediated activation of the *TINF2* promoter in a strong dose-dependent manner (Fig. 5B). A similar effect was seen with pyrrolidine dithiocarbamate (PDTC; Fig. 5C), an antioxidant which also inhibits NF-κB by inhibiting IκB degradation [29]. None of the control vectors (pGL3-Basic, pGL3-Control, or pNFAT-Luc) were impacted by these two NF-κB inhibitors. Similar results were obtained in HEK293 cells transfected with a similar set of plasmids and treated with Bay 11 or PDTC (data not shown). Under similar drug treatment conditions, we found that Mithramycin A and Bay11-7082 also reduced endogenous *TINF2* gene expression levels as compared to those in either untreated cells or cells treated with the vehicle control DMSO (Fig. 5D). Collectively, these studies provide further evidence that Sp1 and NF-κB indeed play important roles in regulating the transcriptional activation of the *TINF2* gene.

## Discussion

Both Sp1 and NF-κB have been shown to be misregulated in the disease state, including various types of human cancer. For example, Sp1 mRNA and DNA-binding activities are increased in epithelial tumors, suggesting that increased activity of this transcription factor contributes to tumor progression in the skin [30], and Sp1 has been shown to be constitutively overexpressed in pancreatic and gastric cancers [31], [32]. Also, Sp1 site-dependent transcription is involved in many signal transduction pathways linked to cancer progression (reviewed in [33]). Constitutive IKK and NF-κB signaling have been implicated in the development of several cancers as well, particularly breast cancer [34], [35], [36], [37]. As maintaining telomeres in order to escape cellular senescence is an important aspect of cancer development and progression, it is possible that alterations in the expression levels and/or activation of these transcription factors could be an early step in a signaling pathway designed to hijack the cellular machinery in order to create the optimal environment for cancer cell growth.

In this study, we have explored the mechanisms that regulate *TINF2* transcription and identified a core promoter region of approximately 406 base pairs, which appears to be at least partially regulated by the Sp1 and NF-κB transcription factors. Our research is in keeping with the finding that Sp1 interacts with components of the transcription machinery to help initiate the transcription of TATA-less promoters, such as the *TINF2* promoter [38], [39], [40]. Furthermore, Sp1 has been implicated in the transcriptional regulation of both core components of human telomerase, hTERT and hTR [41], [42], [43]. Of particular interest is the identification of a functional mutation in a putative Sp1 binding site in the hTR promoter in a patient with paroxysmal nocturnal hemoglobinuria (PNH), a rare blood disorder [43]. As it has been hypothesized that telomere dysregulation is involved in the pathogenesis of several human diseases, including bone-marrow failure disorders (reviewed in [44]) and cancer (reviewed in [45]), future research should be directed toward careful screening of the promoter proximal regions of genes important for telomere maintenance in order to identify any functional sequence changes. In addition, as Sp1 binding sites are GC-rich, there is a strong probability that the transcriptional regulation of these proteins may be subject to epigenetic regulation as well. Therefore, characterization of the methylation status of the endogenous TIN2 promoter may also be warranted.

Teo and colleagues have recently uncovered an unexpected relationship between NF-κB and the shelterin component, hRap1 [25]. They have shown that not only can NF-κB activate transcription of the *hRap1* promoter, but hRap1 itself can positively regulate NF-κB activation through its interaction with IKK. The NF-κB pathway has also been found to be involved in the upregulation of hTERT in HTLV-I-transformed cells [46], as the viral Tax protein can activate NF-κB which in turn stimulates Sp1-dependent transcription [47], [48], a process already known to be important for hTERT regulation. Taking all these data together, it is reasonable to speculate a global regulation mechanism for proteins important for telomere maintenance; however, a detailed exploration of the promoter regions of genes that encode all known components of the telomere binding and maintenance complexes is still necessary.

## Materials and Methods

### Cloning of the TINF2 functional promoter region

Using the available genomic sequence of the *TINF2* gene in the GenBank database (NT 026437), we designed an appropriate primer set (sequences available upon request) to amplify a 2.7 kb DNA fragment corresponding to a region immediately upstream of the known initiation codon of the gene by polymerase chain reaction (PCR). The PCR reaction was performed using the following reaction conditions: 200 ng of genomic DNA isolated from HeLa cells using the DNeasy Tissue Kit (Qiagen), 1U of Phusion Hot-Start DNA Polymerase (FINNZYMES, 2 U/µl), 1×Phusion GC buffer, 0.5 µM primers, 200 µM dNTPs, and 3% v/v DMSO per 50 µl reaction volume. The PCR cycling program was the following: 98°C for 30 sec; 35 cycles of 98°C for 10 sec, 72°C for 90 sec; 72°C for 10 min.

### Generation of luciferase reporter constructs

The 2.7 kb PCR product was digested with HindIII and NcoI (New England Biolabs, NEB), gel-purified (Qiagen), and cloned into the same restriction enzyme sites of the pGL3-Basic vector (Promega) to allow transcription of the firefly luciferase reporter gene under control of this DNA fragment. To generate a series of deletion constructs (Fig. 1A), the plasmid P2731 that contained the 2.7 kb TINF2 promoter sequence was digested with HindIII and either AflII, PvuII, AleI, BIpI, or AvrII, end-polished by the Mung bean nuclease and then self-ligated with T4 DNA Ligase to generate the plasmid constructs P2201, P1668, P553, P450, P351, P248, P148, and P74. All enzymes were purchased from NEB. All other truncation plasmids (Fig. 1B and 1C) were obtained by PCR mutagenesis using the QuickChange method (Stratagene) and appropriate primer sets (sequences available upon request). All plasmid DNAs were confirmed to have the intended sequences by direct sequencing, and their quantity and quality routinely checked by spectrophotometric analysis and agarose gel electrophoresis.

### Luciferase assays in mammalian cells

293T cells were seeded at a density of 4×10^5^ cells per well (or HEK293 cells at a density of 2×10^5^ cells per well) in 12-well plates 24 hours prior to transfection. The cells were transfected with 700 ng of the *TINF2* promoter-driven luciferase plasmid(s) and 100 ng of either the pRSV-β-galactosidase plasmid or the pRL-CMV plasmid as an internal control of transfection efficiency, using Lipofectamine 2000 transfection reagent (Invitrogen) for 293T cells and SuperFect (Qiagen) for HEK293 cells according to the manufacturer's instructions. Cells were harvested 24 hours after transfection and lysed in 200 µl reporter lysis buffer (Promega). Luciferase activity was measured using the Luciferase Assay System (Promega), and β-galactosidase activity was determined by Beta-Glo Assay System (Promega). Luciferase activity (arbitrary units) was divided by the internal control in the same sample to normalize for transfection efficiency and expressed as relative luciferase activity. Transfection data represents at least three independent experiments, each performed in triplicate. The wild-type P450 plasmid construct was also transfected into the Jurkat and K562 human leukemic cells lines using similar conditions as outlined above for 293T cells.

NIH 3T3 p65/p50 double-knockout cells (a kind gift of Dr. David Baltimore at the California Institute of Technology) were maintained in Dulbecco's Modified Minimal Essential Medium (DMEM) with 10% fetal bovine serum under 5% CO_2_ at 37°C. These cells were seeded at a density of 5×10^4^ cells per well in 12-well plates 24 hours prior to being transfected with 500 ng of the appropriate *TINF2* promoter-driven luciferase plasmids, 500 ng of either pCMV4-p50 or pCMV4-p65, or 500 ng of pCMV4 empty vector using SuperFect transfection reagent (Qiagen). Luciferase activities were normalized to the total amount of cellular protein as determined by the Bio-Rad Protein Assay.

### Luciferase assay in Drosophila melanogaster SL2 cells

*Drosophila melanogaster* Schneider SL2 cells, known to lack expression of the Sp1/Sp3 transcription factors, were cultured in HyClone SFX-Insect serum-free medium (HyClone). On the day of transfection, cells were collected, washed once with 1X PBS, and seeded at a density of 1×10^6^ cells/ml in 6-well plates. Cells were then transfected using SuperFect transfection reagent (Qiagen) with 1 µg of the appropriate *TINF2* promoter-driven luciferase reporter plasmid(s) along with varying amounts of pPac-Sp1 plasmid (a kind gift of Dr. Robert Tjian, University of California, Berkeley) or pPac-Sp3 plasmid (a generous gift of Dr. G. Suske, University of Marburg, Germany), and the total amount of DNA brought up to 2 µg per well with the empty pPac plasmid. Forty-eight hours after transfection, cells were harvested in Reporter Lysis Buffer (Promega) and assayed for luciferase activity as suggested by the manufacturer (Promega). Luciferase activities were normalized to the total level of cellular protein as measured by the Bio-Rad Protein Assay.

### Electrophoretic mobility shift assay (EMSA)

Human Sp1 and AP2 proteins were *in vitro* synthesized by the TNT T7 Quick Coupled Transcription/Translation System (Promega) using pcDNA3.1-Sp1 and pcDNA3.1-AP2 (kind gifts of Dr. Ceshi Chen at Emory University) as templates, respectively. Double-stranded DNA oligonucleotides representing the transcriptional binding sites were prepared by denaturing complementary single-stranded oligonucleotides (synthesized by Invitrogen) at 90°C for 10 min and then cooling to room temperature gradually before end-labeling with [γ-^32^P]dATP by T4 polynucleotide kinase (NEB). Oligo sequences are as follows:

412-382 WT: CTACAGCTCCGCTGGGGCGTGGCCTTCTGACG

412-382 Mut: CTACAGCTCCGCTGGAACGTGGCCTTCTGACG

95-59 WT: GTTGCCAGAAGCCCCGCCCCTAGGAGTGATCGGAAAG

95-59 Mut: GTTGCCAGAAGCCCCGTTCCTAGGAGTGATCGGAAAG.

*In vitro* translated protein was incubated in 10 mM Tris-HCl (pH 7.5), 4% glycerol, 1 mM MgCl_2_, 25 mM KCl, 0.5 mM dithiothreitol (DTT), 0.5 mM EDTA, 50 mM NaCl, and 0.5 mg/ml poly(dI-dC) in a final volume of 20 µl for 10 min at room temperature. The incubation was continued for an additional 30 min at room temperature after the addition of 50,000 cpm ^32^P -labeled probes. For competition experiments, 100-fold molar excess of unlabeled DNA oligonucleotides were added to the binding reaction mixture 10 minutes prior to the addition of the labeled probes. For super-shift experiments, 2 µg of antibody against Sp1 (Santa Cruz Biotech) were incubated with the binding reaction mixture on ice for 1 hour before the labeled probe was added. The DNA–protein complexes were separated by electrophoresis on a 4% native polyacrylamide gel in 0.5X TBE at 200 V for 2 hours at 4°C, vacuum-dried, and then autoradiographed.

### Chromatin immunoprecipitation

293T cells in 100 mm cell culture plates (cell number at ∼2.5×10^7^) were cross-linked for 10 min by adding formaldehyde directly into tissue culture medium (final concentration of 1%) at room temperature with mild shaking. The reaction was stopped by adding glycine (125 mM), and the cells were kept at room temperature for 5 minutes. The cross-linked cells were then washed twice with cold PBS, scraped, pelleted, and resuspended in 600 µl of Nuclei Lysis Buffer (50 mM Tris-HCl, pH 8.1, 10 mM EDTA, 1% SDS) with protease inhibitors (1 mM PMSF, 10 µg/ml leupeptin, 1 µg/ml pepstatin A, 1 µg/ml aprotinin) (Sigma). The lysates were then sonicated for four cycles of 10 sec each, resting on ice for 2 min between cycles, on a Branson Sonifier 450 (settings: duty cycle  = 50%; output control  = 3), resulting in chromatin fragmentation to an average length of 500–1000 bps. After sonication, the samples were then centrifuged at 16,000 *g* for 10 minutes. Sheared chromatin was diluted 20-fold in ChIP dilution buffer (0.01% SDS, 1% Triton X-100, 1.2 mM EDTA, 16.7 mM Tris-HCl, pH 8.1, and 167 mM NaCl) with protease inhibitors as described above. Then, 4 µg of Rabbit anti-Sp1 antibody (Santa Cruz Biotech, PEP 2), Rabbit anti-p65 antibody (Santa Cruz Biotech, C-20), control antibody (Rabbit anti-c-Myc, Santa Cruz Biotech, A14), or no antibody was added to each aliquot of chromatin extract and the reaction mixture incubated overnight at 4°C on a rotary shaker. Complexes were captured by incubation with protein A/G agarose beads (Santa Cruz Biotech) blocked with 250 µg/ml of sheared salmon sperm DNA (Ambion) and 1 mg/ml BSA (NEB) in ChIP dilution buffer at 4°C for 4 hours. Captured complexes were washed successively with ChIP dilution buffer, high-salt wash buffer (0.1% SDS, 1% Triton X-100, 2 mM EDTA, 20 mM Tris-HCl, pH 8.1, 500 mM NaCl), LiCl wash buffer (0.5 M LiCl, 1% NP40, 1% deoxycholate, 100 mM Tris-HCl, pH 8.1), and TE buffer (10 mM Tris-HCl, pH 8.0, 1 mM EDTA). Complexes were washed twice in each buffer for 10 min apiece with shaking and then centrifuged to collect protein A/G agarose beads. After the final wash, 250 µl of elution buffer (1% SDS, 0.1 M NaHCO_3_) was added and incubated at room temperature for 15 minutes with rotation. Then, 5 M NaCl was added to reverse the formaldehyde cross-linking by heating at 65°C for 4 hours. After precipitation with ethanol, the pellets were resuspended and treated with proteinase K (NEB). DNA was recovered by standard phenol–chloroform extraction and ethanol precipitation. Pellets were resuspended in TE buffer and subjected to polymerase chain reaction (PCR) amplification using the following primers to amplify the TIN2 fragment: Forward (5′-CTTCTGACGCACCGTCACGG-3′) and Reverse (5′-CACCAGGGGCGTAGCCATGG-3′). The PCR products were separated by agarose gel electrophoresis.

### Generation of luciferase reporter plasmids carrying point mutations

Transcriptional binding site mutant plasmids (Fig. 4A and 4B) were generated in the minimal promoter pGL3-P406 backbone by PCR mutagenesis using the QuickChange method (Stratagene) and appropriate primer sets (sequences available upon request). Sequences of the putative binding site mutants are as follows:

P406 (397−396) WT: GCTGGGGCGTGG;

P406 (397−396) Mut: GCTGGAACGTGG;

P406 (79−78) WT: CCCCGCCCCTAG;

P406 (79−78) Mut: CCCCGTTCCTAG;

P406 (99−96) WT: CGACAGGGAGTTGC;

P406 (99−96) Mut: CGACAAAATGTTGC;

P406 (64−60) WT: TGATCGGAAAGCCTC;

P406 (64−60) Mut: TGATCAACCCGCCTC. Numbers in parentheses denote mutated nucleotides, which are underlined. All mutated DNAs were confirmed by direct sequencing, and their quantity and quality routinely checked by spectrophotometric analysis and agarose gel electrophoresis.

### Pharmacological inhibitors

293T or HEK293 cells were seeded at a density of 2×10^5^ cells per well in 12 well plates 24 hours prior to transfection. Bay11-7082 (Sigma) at 100 nM or 10 µM concentration, PDTC (Sigma) at 500 nM or 5 µM, or Mithramycin A (Sigma) at 1 nM or 100 nM was added directly to the media 1 hour prior to being transfected with 1 µg of either the minimal promoter construct (pGL3-P406), pGL3-Basic, pNFAT-Luc, or pGL3-Control along with 70 ng of pRL-CMV using Lipofectamine 2000 (Invitrogen) for the 293T cells and SuperFect (Qiagen) for the HEK293 cells according to the manufacturers' instructions. Media was changed 4 hours post-transfection to a fresh aliquot of the media that contain the same amounts of the chemicals as shown above. 24 hours later, cell lysates were prepared for luciferase activity measurements using the Dual-Luciferase Reporter Assay System (Promega). Firefly luciferase reporter readings were normalized to the co-transfected *Renilla* luciferase values and expressed as relative luciferase activity. All reactions were done in triplicate.

HEK293 cells were seeded at a density of 5×10^5^ cells per well in a 6-well format. Bay11-7082 (Sigma) at 10 µM concentration or Mithramycin A (Sigma) at 100 nM was added directly to the media. As controls, cells were either treated with DMSO at 10 µM concentration used to dissolve the compounds or left untreated. Twenty-four hours later, total RNA was extracted from cells using Bee reagent (Tel. Test Inc.) and quantified. Fifty micrograms of each of samples was treated with RNase-free DNase I (Invitrogen) for 30 minutes at 37°C followed by its inactivation at 85°C for 20 minutes. Thirty micrograms of each sample was used for reverse transcription using the Superscript III reverse transcriptase as described by the manufacturer (Invitrogen). Oligo dT and GAPDH primers (sequences available upon request) were used in the reverse transcription reaction at 50°C for 1 hr. The quantitative real-time PCR was carried out in a 20 ul reaction mixture containing primers specific for the TINF2 gene (Fd: 5′-GGAGTTTCTGCGATCTCTGC-3′, Rv: 5′ GTTTCCTGTGCCTCCAAAATC-3′) or for the GAPDH RNA (Fd: 5′-GAAGGT GAAGGTCGGAGTC-3′ and Rv: 5′-GAAGATGGTGATGGGATTTC-3′) by using Sybr green DNA dye (Invitrogen) in the reaction mixture. The PCR conditions were 50°C for 2 min, 95°C for 2 min, and 45 cycles of 95°C for 15 s, 55°C for 30 s, and 72°C for 30 s. The TINF2 RNA levels, expressed as threshold cycle (*cT*) values, were normalized to the GAPDH RNA levels.

### Statistical analysis

All statistical analyses were performed using a two-tailed Student's t test. *p<0.0.5, **p<0.01, ***p<0.001
